# Supplementary material for: EGF and IgA in maternal milk, donor milk, and milk fortifiers in the neonatal intensive care unit setting
Source: PLoS One. 2025 Apr 16;20(4):e0313465. doi: 10.1371/journal.pone.0313465 (PMC12002475; doi:10.1371/journal.pone.0313465)
Supplement: Attachment — Submitted filename: Review.docx [file pone.0313465.s001.docx]

This is a prospective cohort study comparing EGF and IgA concentrations as important infant health markers, in various sources of milk for premature infants.

The aim behind the study is clear and the significance of the study outcomes may help contribute towards decision making in NICU around premature infant nutrition. However, there are a number of missing information and methodological vagueness that needs to be addressed:

- One of the main objectives of the study was to analyze milk fortifiers. However, very little information is provided about what these are and how they are used in NICU. More information needs to be provided in the introduction and method section
  - The concentration of EGF in the HM fortifier is around 100ng/ml and yet when it was added to the maternal milk, it only increased EGF by 20ng/ml. What was the dilution factor for maternal milk to fortifier? This needs to be described in the methods section. And can’t one simply add more of the fortifier if needed?
  - Other information that needs to be provided includes what is the shelf life of fortifiers? How easy is it to get them and is there realistically enough to be used in every NICU?
- Have the authors recorded the age from parturition of the individual milk donated? This may explain the difference between the pooled and individual milk concentration of EGF and IGA. If it hasn’t been recorded, an explanation to why that is needs to be provided
- Which infant formulas have been used? There are many kinds and at the very least there needs to be a discussion about how the infant formula used in this NICU may differ in formulation from other formulas out there
- There were 74 participants and 237 samples. Does that mean that an individual infant would have had different diets at different times? For example, can one infant be given maternal milk one day and formula the other day? If so, has this been recorded, and could it have affected the results from the stool samples?
- In the first section, maternal milk has 59.72 ng/ml EGF, in the other section it is 49.11 ng/ml EGF. Why is it different? Did the authors measure before and after fortification in the second section? If so, this needs to be described clearly in the methods section. Similarly for IGA.
- More information needs to be provided about how each diet is received, where it is sourced from and prepared for the infant.
- For pearson correlations, an R=0.1885 is not significant.

Other points:

- P-values should be provided in the text of the results section and abstract.
- The methodology section needs re-writing to be more organized and detailed.
- Is there a reason why stool was not analyzed for IGA?
- “Our observed correlation between EGF and IgA concentrations in a given milk sample suggests that inclusion of individual donor milk highly concentrated in one milk fact within a donor pool may increase the concentration of other beneficial factors in the final pooled donor milk.”---Not clear what this means
- Translocation to where?
- In the introduction, authors mentioned a pre-clinical model regarding milk expressed closer to parturition. This needs to be expanded on the type of model used.
- Need to define what “diet” means for the purpose of the study. Since both mum and infant were recruited in the study. Similarly, define what “participant” refers to in the study.
- “Clinical data was de-identified”. When was it de-identified and who was blinded to this information?
- “Incomplete sample pairs were discarded”. More information needs to be provided here. What does that mean? Who made the decision to discard?
- The small para about IgA bacterial binding in the methods sections, needs to be expanded and more information about the method provided clearly.
- How was the poop samples homogenized?
- The article could do with an editorial review to correct grammatical errors throughout.
